# Rikkunshi-to attenuates adverse gastrointestinal symptoms induced by fluvoxamine

**DOI:** 10.1186/1751-0759-1-21

**Published:** 2007-11-15

**Authors:** Takakazu Oka, Yoko Tamagawa, Sota Hayashida, Yuko Kaneda, Naoki Kodama, Sadatoshi Tsuji

**Affiliations:** 1 Division of Psychosomatic Medicine, Department of Neurology, University of Occupational and Environmental Health, Iseigaoka 1-1, Yahatanishi-ku, Kitakyushu, 807-8555, Japan

## Abstract

**Background:**

Upper gastrointestinal (GI) symptoms such as nausea and vomiting are common adverse events associated with selective serotonin reuptake inhibitors (SSRIs), and may result in discontinuation of drug therapy in patients with depressive disorder. Rikkunshi-to (formulation TJ-43), a traditional herbal medicine, has been reported to improve upper GI symptoms and comorbid depressive symptoms in patients with functional dyspepsia. The aim of the present study was to determine if TJ-43 reduces GI symptoms and potentiates an antidepressant effect in a randomized controlled study of depressed patients treated with fluvoxamine (FLV).

**Methods:**

Fifty patients with depressive disorder (19–78 years, mean age 40.2 years) were treated with FLV (n = 25) or FLV in combination with TJ-43 (FLV+TJ-43) (n = 25) for eight weeks. The following parameters of the two groups were compared: The number of patients who complained of adverse events and their symptoms; GI symptoms quality of life (QOL) score, assessed by the Gastrointestinal Symptom Rating Scale (GSRS), Japanese edition, before and two weeks after beginning treatment; and depressive symptoms assessed by the Self-Rating Depression Scale (SDS), before and 2, 4, and 8 weeks after beginning treatment.

**Results:**

The number of patients who complained of adverse events in the FLV+TJ-43 group (n = 6) was significantly lower than the number complaining in the FLV group (n = 13) (*P *< 0.05). The number of patients who complained of nausea was also lower in the FLV+TJ-43 group (n = 3) than in the FLV group (n = 9) (*P *< 0.05). By two weeks after treatment, GSRS scores had improved in the FLV+TJ-43 group, but not in the FLV group. SDS scores were not different between the two groups at any of the assessment points.

**Conclusion:**

This study suggests that Rikkunshi-to reduces FLV-induced adverse events, especially nausea, and improves QOL related to GI symptoms without affecting the antidepressant effect of FLV.

## Background

Upper gastrointestinal (GI) symptoms such as nausea and vomiting are one of the most common adverse events caused by selective serotonin reuptake inhibitors (SSRIs) [1-6]. In some patients, upper GI symptoms are critical issues that impair their quality of life (QOL) and may result in discontinuation of SSRI therapy.

Rikkunshi-to is a traditional Japanese herbal (Kampo) medicine for treating upper GI tract symptoms such as nausea, indigestion, and anorexia. Rikkunshi-to Extract Granules for Ethical Use (Tsumura and Co., Product number TJ-43) (7.5 g), containing 4.0 g of dried extract obtained from mixed raw herbs in the following ratio: JP Atractylodes Lancea Rhizome, 4.0 g; JP Ginseng, 4.0 g; JP Pinellia Tuber, 4.0 g; JP Poria Sclerotium, 4.0 g; JP Jujube, 2.0 g; JP Citrus Unshiu Peel, 2.0 g; JP Glycyrrhiza, 1.0 g; and JP Ginger,0.5 g; has been approved for medicinal use by the Japanese Ministry of Health and Welfare. Today in Japan, Rikkunshi-to is widely used for treating the upper GI symptoms of patients with functional dyspepsia (FD) [7,8] and gastroesophageal reflux [9], dyspeptic symptoms of postgastrointestinal surgery patients [10], and chemotherapy-induced nausea in breast cancer patients [11]. Rikkunshi-to has also been reported to improve depressive symptoms and high cortisolemia in depressed FD patients [12,13].

We therefore hypothesized that Rikkunshi-to might attenuate SSRI-induced GI symptoms and potentiate the antidepressant effect of SSRIs. To test this hypothesis, a randomized controlled study was done in which we treated depressed patients with fluvoxamine maleate (FLV) with or without Rikkunshi-to, and compared the rates of adverse reactions of the two groups, focusing on GI symptoms and changes in self-rating depression scores.

## Subjects and methods

### Subjects

This study was approved by the Ethics Committee of the University of Occupational and Environmental Health, Japan, and conducted in accordance with its guidelines.

Fifty patients with depressive disorders were randomly assigned to one of the following groups: FLV group (n = 25) or FLV plus Rikkunshi-to (FLV+TJ-43) group (n = 25). The subjects were treated with either FLV or FLV plus TJ-43 for eight weeks. They were all diagnosed according to the Diagnostic and Statistical Manual of Mental Disorders IV as having depressive disorders, including major and minor depressive disorders. In both groups, the initial daily dose of FLV was 50 mg, and the dose was increased weekly up to 150 mg. The dose of FLV was adjusted in accordance with a patient's adverse reactions or at their request. Therefore, the final maximal dose in some patients was less than 150 mg. The FLV+TJ-43 group received a granular extract of Rikkunshi-to (TJ-43, Tsumura and Co., Tokyo, Japan) at a daily dose of 7.5 g (2.5 g orally, three times per day). Subjects who had taken any antidepressants, prokinetics, or herbal medicines were excluded from this study. However, subjects who were taking hypnotics or benzodiazepines regularly for more than two weeks before the study were included, and the doses of those drugs were not changed during the investigation period.

Some patients complained of GI symptoms as well as depressed mood before the start of the treatment. These cases underwent an upper GI endoscopy, colorectal endoscopy, or ultrasonography to confirm that there were no organic diseases accounting for their GI symptoms. Patients in whom organic diseases such as gastric ulcer were found were excluded from this study even though they were depressed. Patients complaining of severe GI symptoms who asked for symptomatic treatment as well as antidepressants were excluded from this study, even if there were no organic lesions detected and they had been diagnosed with a depressive disorder. The only subjects enrolled in this study were those whose GI symptoms were not so severe that they required symptomatic relief before the start of the treatment.

### Assessment

The following parameters were compared between the FLV group and the FLV+TJ-43 group: (1) the number of patients who complained of adverse events and their symptoms; (2) GI symptoms specific QOL score according to the Gastrointestinal Symptom Rating Scale (GSRS), Japanese edition [14]; and (3) depressive symptoms according to the Self-rating Depression Scale (SDS), Japanese edition [15,16]. The GSRS scores were also compared in each group between the pretreatment and posttreatment periods.

Adverse events were checked during interviews by physicians at every patient visit, which took place before and 1, 2, 4, 6, and 8 weeks after the start of the treatment. Patients were also instructed to call their physicians if they experienced adverse events, because previous studies have demonstrated that FLV-induced adverse events occur within one or several days after taking FLV. In this study, we listed the symptoms as adverse events if the patient described them as adverse events. As described in the Results, a considerable number of patients had GI symptoms before the start of the treatment. Therefore, if patients found it difficult to distinguish GI adverse events from daily fluctuations of depression-related GI symptoms, those events were not listed as adverse. Whenever subjects complained of adverse events and asked for alleviation of their symptoms, they were treated symptomatically with antiemetic drugs or laxatives. Patients requesting symptomatic relief during an evaluation visit were excluded from GSRS and SDS assessments after that visit.

The GSRS is a self-administered questionnaire for assessing GI symptoms, and consists of 15 items [14,17,18]. These include five subscales for reflux, abdominal pain, indigestion, diarrhea, and constipation. The higher the scores, the more pronounced the symptoms. GSRS total scores and the five subscale scores were compared before and 15 days after beginning treatment in patients who were able to take FLV continuously for two weeks without asking for any medications to treat adverse events. GSRS was not assessed two weeks after treatment in those who complained of GI adverse events if they had already stopped taking FLV or FLV+TJ-43, or if they had already been treated symptomatically before the post-treatment GSRS assessment.

SDS scores between the FLV group and the FLV+TJ-43 group were compared at time points before and 2, 4, and 8 weeks after treatment.

### Statistical analyses

The Student *t*-test for unmatched data, Welch t test, and Mann-Whitney U test were used. The Fisher exact probability test was also used when appropriate.

The paired *t *test was used to compare GSRS scores before and after treatment. Statistical significance was considered to be *P *< 0.05.

## Results

### Demographic and baseline characteristics

There were no differences in the demographic and baseline characteristics between the FLV and the FLV+TJ-43 groups for age, sex, GSRS total and five subscale scores, and SDS score (Table 1). Upper GI symptoms seen before the start of the treatment were epigastral discomfort (8 patients in the FLV group vs. 10 patients in the FLV+TJ-43 group), nausea (7 patients vs. 11 patients, respectively) and abdominal fullness (5 patients vs. 4 patients, respectively). There were no between group differences in the incidence of GI symptoms or the administered dose of FLV at any time point (Table 2).

**Table 1:** Patient characteristics of the treatment groups.

Variable	FLV	FLV+TJ-43	
Number	25	25	
Age, years	40.3 ± 17.3	40.2 ± 16.1	n.s.^1)^
Sex			
Male	18 (72%)	18 (72%)	
Female	7 (28%)	7 (28%)	n.s.^2)^
GSRS total score	2.30 ± 1.18	2.53 ± 1.03	n.s.^3)^
SDS score	56.0 ± 7.0	56.6 ± 7.8	n.s.^1)^

FLV = fluvoxamine; TJ-43 = Rikkunshito; GSRS = gastrointestinal symptom rating scale; SDS = self-rating depression scale. mean ± SD. N.s. = not significant.

1) Student *t*-test for unmatched data. 2) Fisher exact probability test. 3) Student *t*-test for unmatched data and Mann-Whitney U test.

**Table 2:** Mean dose of FLV.

Time	FLV (n)	FLV+TJ-43 (n)	
1^st ^week	48.9 ± 5.1 (25)	48.0 ± 6.9 (25)	n.s.
2^nd ^week	85.3 ± 22.6 (19)	92.1 ± 18.7 (19)	n.s.
4^th ^week	125.0 ± 35.0 (13)	134.2 ± 33.5 (19)	n.s.
8^th ^week	129.2 ± 32.5 (13)	136.1 ± 33.5 (19)	n.s.

In parentheses, number of patients is indicated. FLV = fluvoxamine; TJ-43 = Rikkunshito. mean ± SD. N.s. = not significant. Student *t*-test for unmatched data.

### Adverse events

**Figure 1:**
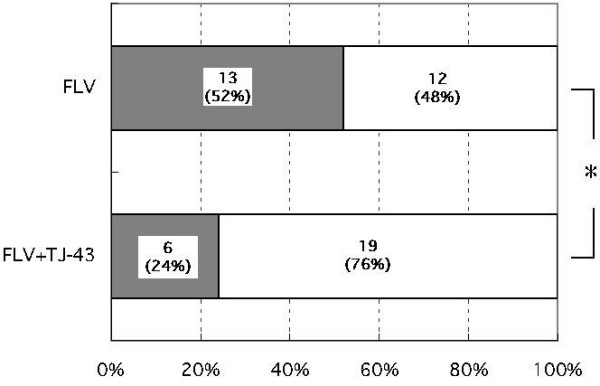
The number of patients who complained of adverse events. Percentage is shown in parentheses. *P < 0.05 by the Fisher exact probability test. FLV = fluvoxamine; TJ-43 = Rikkunshi-to. The number (%) is shown in the column.

13 out of 25 patients (52%) in the FLV group and 6 out of 25 patients (24%) in the FLV+TJ-43 group complained of adverse events. The incidence of adverse events in the FLV+TJ-43 group was significantly lower than in the FLV group (*P *< 0.05, Fig. 1). Adverse reactions are listed in Table 3. In the FLV group, the percentages of patients complaining of GI symptoms that were considered adverse events, were nausea, 36%; anorexia, 12%; abdominal fullness, 4%; constipation, 4%; and diarrhea, 4%. In the FLV+TJ-43 group, the percentages of subjects complaining of symptoms considered adverse events were nausea, 12%, and anorexia, 12%. The number of subjects who complained of nausea in the FLV+TJ-43 group was significantly lower than in the FLV group (*P *< 0.05, Table 3). As previously described, only the symptoms described by patients as not being daily fluctuations of depression-related GI symptoms were listed as adverse events (Table 3). Patients did not seem to have difficulty in distinguishing between adverse events and daily fluctuations, because there were differences in severity and the time of occurrence of symptoms, e.g. more severe nausea than usual occurring within several hours after taking the first FLV tablet. In both groups, most adverse events appeared just after beginning treatment or within several days after increasing the dose of FLV. They appeared 1–15 days (7.9 ± 4.9 days, mean ± SD) after beginning treatment in the FLV group and 1–8 days (5.6 ± 2.6 days) after beginning treatment in the FLV+TJ-43 group. Because of adverse reactions, 12 subjects in the FLV group and 6 subjects in the FLV+TJ-43 group asked for medication or stopped taking FLV during the investigation period. One subject in the FLV group complained of discomfort one week after beginning FLV (50 mg), but asked to continue the FLV therapy. This patient was treated with FLV for eight weeks without increasing the dose.

**Table 3:** Adverse events associated with FLV and FLV+TJ-43 treatment.

	FLV	FLV+TJ-43	
		
Adverse events	Number (%) of patients reporting event		
GI symptoms			
Nausea	9 (36)	center3 (12)	*
Anorexia	3 (12)	3 (12)	
Abdominal fullness	1 (4)	0 (0)	
Constipation	1 (4)	0 (0)	
Diarrhea	1 (4)	0 (0)	
Feel discomfort	3 (12)	2 (8)	
Feel irritable	1 (4)	0 (0)	
Difficulty in urinating	1 (4)	0 (0)	
General fatigue	1 (4)	0 (0)	
Tremor	0 (0)	1 (4)	

	/25	/25	

FLV = fluvoxamine; TJ-43 = Rikkunshito; GI:gastrointestinal. * p < 0.05 by Fisher exact probability test.

### GI symptoms

The GSRS total and five subscale scores were compared between the FLV group and the FLV+TJ-43 group at pretreatment (Table 4). There were no between group differences in any of the GSRS scores at baseline. The pretreatment scores and 15 day scores were then compared for those who took FLV or FLV+TJ-43 continuously for two weeks. In the FLV group (n = 15), there were no differences in the means of the GSRS total and five subscale scores between the pretreatment and posttreatment periods. In the FLV+TJ-43 group (n = 19), the mean of the GSRS total scores significantly improved from 2.45 to 1.97 (*P *< 0.05). Among the five subscale scores, the mean GSRS abdominal pain score showed a tendency to improve, from 3.03 to 2.18, and the mean GSRS diarrhea symptom score showed a tendency to improve, from 2.25 to 1.65, following treatment.

**Table 4:** Changes in GSRS scores by FLV and FLV+TJ-43 treatment.

GSRS score	pre-treatment	post-treatment	
Total			
FLV	2.42 ± 1.24	2.52 ± 0.99	n.s.
FLV+TJ-43	2.45 ± 1.10	1.97 ± 0.81	*
Reflux			
FLV	2.13 ± 1.46	2.50 ± 1.35	n.s.
FLV+TJ-43	2.37 ± 1.61	2.00 ± 0.91	n.s.
Abdominal pain			
FLV	2.63 ± 1.56	3.18 ± 1.81	n.s.
FLV+TJ-43	3.03 ± 1.82	2.18 ± 1.00	+
Indigestion			
FLV	1.70 ± 0.97	1.87 ± 0.74	n.s.
FLV+TJ-43	2.05 ± 0.95	1.78 ± 1.06	n.s.
Diarrhea			
FLV	2.87 ± 1.78	2.35 ± 1.60	n.s.
FLV+TJ-43	2.25 ± 1.47	1.65 ± 1.06	+
Constipation			
FLV	2.64 ± 1.87	2.83 ± 1.33	n.s.
FLV+TJ-43	2.66 ± 1.69	2.34 ± 1.53	n.s.

FLV = fluvoxamine; TJ-43 = Rikkunshito. mean ± SD. * p < 0.05, + p < 0.1 by paired *t*-test.

### Depressive symptoms

**Figure 2:**
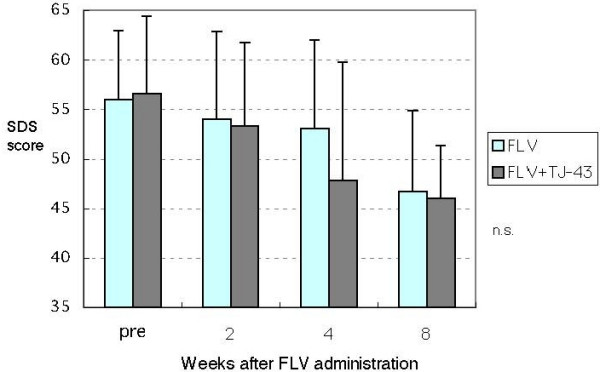
Changes in SDS scores. FLV = fluvoxamine; TJ-43 = Rikkunshi-to. Not significant at any time point by Student *t*-test for unmatched data, Welch t test, and Mann-Whitney U tests.

SDS scores were compared between the FLV group and the FLV+TJ-43 group before and 2, 4, and 8 weeks after the start of treatment. There were no between group differences in the SDS scores at any time point (Fig. 2). Although the mean SDS scores four weeks after the beginning of treatment were 53.1 in the FLV group (n = 13) and 47.8 in the FLV+TJ-43 group (n = 19), the difference was not statistically significant.

## Discussion

This study demonstrates that the number of subjects who reported adverse events was significantly lower in the FLV+TJ-43 group than in the FLV group, suggesting that Rikkunshi-to attenuates SSRI-induced adverse reactions. In the present study, 52% of the patients who took FLV complained of one or more adverse events. This incidence is similar to those seen in previous clinical trials conducted in Japan, in which 47–51% of subjects reported adverse events when they took FLV at 90–150 mg daily [2-4]. By contrast, only 24% of patients in the FLV+TJ-43 group complained of adverse events. Nausea was the most common adverse event of FLV, and 10–37% of subjects who took FLV reported nausea in previous studies [2-6]. In this study, 36% of the patients in the FLV group complained of nausea, contrasting with only 12% of the patients in the FLV+TJ-43 group who complained of nausea, a significantly lower incidence. Rikkunshi-to also has been reported to have adverse reactions. Typical adverse events of Rikkunshi-to are diarrhea and edema. Previously reported incidences of diarrhea and edema were 0–2.3% and 0–6.7%, respectively [7,8,12]. In this study, no patient in the FLV+TH-43 group reported diarrhea or had edema. Therefore, Rikkunshi-to may reduce the incidence of FLV-induced nausea without causing the reported adverse reactions.

Rikkunshi-to is a traditional herbal (Kampo) prescriptions approved by Japanese National Health Insurance for medicinal use (for review, see [19]). It has been widely prescribed to treat upper GI symptoms in patients with FD [7,8,12], gastroesophageal reflux [9], dyspeptic symptoms of children after surgery [10], and chemotherapy-induced nausea in breast cancer patients [11]. A double-blind, randomized, controlled study has demonstrated the usefulness of Rikkunshi-to for FD [8]. Another study demonstrated that Rikkunshi-to is more effective than the prokinetic drug, cisapride [7]. The present study suggested that Rikkunshi-to is also effective for SSRI-induced nausea.

Rikkunshi-to is well known for improving dysfunction in gastric motility, which may cause upper GI tract symptoms. It has been demonstrated to promote gastric adaptive relaxation in isolated guinea pig stomachs [20], and to facilitate delayed gastric emptying in rats [21] and in FD patients [22,23]. Rikkunshi-to has also been demonstrated to prevent intracellular signaling disorders in the gastric smooth muscle of diabetic rats [24].

The precise mechanisms of FLV-induced nausea remain uncertain. However, stimulation of serotonin (5-HT)3 receptors in the enteric nervous system or within the brain may cause SSRI-induced nausea, because 5-HT3 receptor antagonists such as ondansetron [25] and cisapride [26] have been reported to attenuate SSRI-induced nausea. Furthermore, it is suggested that FLV-induced nausea is associated with serotonergic neuronal hyperactivity in the GI tract [6]. Thus far it is not known if Rikkunshi-to affects serotonergic systems in the enteric nervous system or within the brain; however, the effects of Rikkunshi-to on gastric function may be attributed, at least in part, to its inhibitory effect on SSRI-induced GI symptoms and its alleviation of GI tract symptoms related to QOL.

We previously demonstrated that administration of Rikkunshi-to for four weeks decreased depressive symptoms in FD patients, especially general fatigue, depressed mood, and loss of taste [12]. Therefore, we were very interested in seeing if Rikkunshi-to could potentiate the antidepressant effect of FLV. There were no differences in the mean SDS scores between the FLV and FLV+TJ-43 groups at two and eight weeks. The mean SDS scores at four weeks were lower in the FLV+TJ-43 group than in the FLV group; however, the difference was not statistically significant. These results may be partly because of the relatively small number of subjects studied. Therefore, an additional larger study is required to determine if Rikkunshi-to really exhibits an additive antidepressant effect in addition to FLV. However, from a different perspective, this study clearly showed that Rikkunshi-to does not inhibit the antidepressant effect of FLV.

Also limiting the study is that it was conducted by comparing effects seen in an FLV group and an FLV+TJ-43 group. To avoid bias, however, comparisons might better have been made between an FLV+placebo group and an FLV+TJ-43 group.

## Conclusion

This study suggests that Rikkunshi-to attenuates SSRI-induced nausea and improves QOL related to GI symptoms, without affecting the antidepressant effect of fluvoxamine.

## Competing interests

The author(s) declare that they have no competing interests.

## Authors' contributions

TO designed the study protocol, analyzed the data, and drafted the manuscript. TO, YT, SH, YK, and NK conducted the study. ST supervised the study. All authors read and approved the final manuscript.
